# Colonization of bacterial and viral respiratory pathogens among healthcare workers in China during COVID–19 pandemic

**DOI:** 10.1080/20002297.2024.2365965

**Published:** 2024-06-20

**Authors:** Dandan Yang, Jianan Xu, Tao Wu, Wei Zhang, Xiaojun Zhu, Zhengdong Zhang, Baoli Zhu

**Affiliations:** a Department of Environmental Genomics, Jiangsu Key Laboratory of Cancer Biomarkers, Prevention and Treatment, Collaborative Innovation Center for Cancer Personalized Medicine, School of Public Health, Nanjing Medical University, Nanjing, PR China; b Department of Sexually Transmitted Diseases and AIDS, Center for Disease Control and Prevention of Jiangsu Province, Nanjing, PR China; c Department of Genetic Toxicology, The Key Laboratory of Modern Toxicology of Ministry of Education, Center for Global Health, School of Public Health, Nanjing Medical University, Nanjing, PR China; d Department of Policy Research Office, Center for Disease Control and Prevention of Jiangsu Province, Nanjing, PR China; e Department of Pathogenic Microbiology, Center for Disease Control and Prevention of Jiangsu Province, Nanjing, PR China; f Department of Public Health, Kunshan Hospital of Traditional Chinese Medicine, Suzhou, PR China; g Department of Prevention and Health, The Second Affiliated Hospital of Soochow University, Suzhou, PR China

**Keywords:** Respiratory infections, healthcare workers, occupational factors, preventive measures, COVID-19

## Abstract

**Background:**

Healthcare settings may amplify transmission of respiratory pathogens, however empirical evidence is lacking. We aimed to describe the spectrum and distribution of respiratory pathogens among healthcare workers in eastern China.

**Methods:**

Healthcare workers were recruited from October 2020 to November 2021 in Jiangsu province. Participants were interviewed regarding demographic and hospital-based protective measures. Thirty-seven common respiratory pathogens were tested using real-time PCR/RT-PCR (Probe qPCR). The role of demographic and hospital-based protective measures on pathogens colonization using multivariable logistic regression models.

**Results:**

Among 316 enrolled healthcare workers, a total of 21 pathogens were detected. In total, 212 (67.1%) healthcare workers had at least one respiratory pathogen; 195 (61.7%) and 70 (22.2%) with a bacterial and viral pathogen. The most commonly detected pathogen was streptococcus pneumoniae (47.5%) followed by *Haemophilus* influenzae (21.2%). One hundred and five (33.2%) healthcare workers with copathogens had at least two respiratory pathogens. Both bacterial and viral colonization were more common in 2020 compared to 2021. A decreased risk of colonization was seen in participants with infection prevention and control training and suitable hand hygiene.

**Conclusions:**

Colonization of respiratory pathogens in healthcare workers from eastern China was high. Differential risk was impacted only by hospital-based protective measures and not demographic factors.

## Introduction

Healthcare settings are important amplifiers of transmission [1–3]. Close proximity of persons and handling of human secretions enhance the risk for transmission [4]. Healthcare workers are at frontline during epidemics and pandemics, and protecting healthcare workers has received significant attention in recent years during outbreaks of emerging and re-emerging infectious diseases [5,6]. WHO has issued recommendations on the rational use of personal protective equipment (PPE) in hospital and community settings [7]. Nevertheless, protecting healthcare workers remains a challenge for most countries, where shortages of adequate PPE are a daily concern [8]. Limited testing capacity precludes early identification and isolation of cases, leading to unnecessary occupational exposure for healthcare workers. While social distancing measures require most people to stay home, healthcare workers face the opposite reality [9]. Increased patient loads due to the COVID-19 pandemic have resulted in longer working hours, putting them at a heightened risk of contracting the virus.

Compared to control (ie, no use of corresponding PPE item), use of gloves, gowns, surgical masks, N95 respirators, and face protection were associated with large reductions in the risk of infection [10]. Recommended infection prevention and control (IPC) measures by WHO include hand hygiene, medical mask, use of PPE, single or cohort patients, sterilization of patient-care equipment and linen, etc. [11]. However, the work stress and risk of infection faced by healthcare workers remain high, particularly in health systems already grappling with workforce shortage due to lack of trained personnel, skilled labor migration and geographical maldistribution, even prior to pandemic times [12,13]. Although various guidelines and policies for IPC are implemented in healthcare settings worldwide, the risk of transmission of respiratory infectious diseases in Chinese healthcare workers has not been previously investigated.

To fill this knowledge gap, we aimed to investigate colonization and the spectrum of respiratory pathogens in healthcare workers from eastern China during the COVID-19 pandemic. We also assessed whether specific demographic and/or hospital-based protective measures were associated with viral and/or bacterial colonization.

## Methods

### Study population

We recruited healthcare workers (including doctors, nurses, medical technicians, administrative and labor personnel) in Kunshan Hospital and the Second Affiliated Hospital of Suzhou University from October 2020 to November 2021. Inclusion criteria were as follows [1]: healthcare workers who work in Kunshan Hospital and the Second Affiliated Hospital of Suzhou University for more than 1 year [2]; use PPE in daily work [3]; have not taken antibiotics within 2 weeks of the collection of pharyngeal swabs [4]; and no respiratory tract infection symptoms. Exclusion criteria included off-duty personnel during the investigation and refusing to participate in the study.

### Questionnaire investigation

We designed a structured questionnaire to collect the PPE and IPC measures of healthcare workers. The main content included demographic data, monthly income, place of daily work, occupation type, daily working time, information of PPE (including type and replacement frequency of protective equipment), methods of ventilation and disinfection in workplace, IPC training, hand hygiene training, occupational exposures and injury, hand hygiene and implementation.

### Laboratory tests

Nucleic acid was extracted by nucleic acid extraction kit (EX-DNA/RNA Virus) (Tianlong Technology Company, Xi’an, China). For PCR amplification, we used One Step PrimeScript™ RT-PCR Kit/Premix EX Taq™ (Probe qPCR) (TaKaRa Company, Dalian, China).

Total DNA/RNA of bacteria and virus was extracted according to the protocol of automatic nucleic acid extraction instrument and nucleic acid extraction kit. One Step PrimeScript™ RT-PCR/Premix Ex Taq™ (Probe qPCR) Kit was used to detect 37 common respiratory pathogens in samples by real-time PCR/RT-PCR [14]. These included influenza A virus, influenza B virus, human metapneumovirus, human Boca virus, human adenovirus, herpes simplex virus (type 1 and 2), cytomegalovirus, Epstein–Barr virus, respiratory syncytial virus, parainfluenza virus (type 1–4), human coronavirus (type 229, OC43, NL63, and HKU), rhinovirus, *Mycoplasma pneumoniae*, *Chlamydia pneumoniae*, *Legionella pneumophila*, *Streptococcus pneumoniae*, *Haemophilus influenzae*, *Moraxella catarrh*, *Klebsiella pneumoniae*, Mycobacterium tuberculosis, *Acinetobacter baumannii*, *Pseudomonas aeruginosa*, *Escherichia coli*, *Staphylococcus aureus*, *Bordetella pertussis*, *Aspergillus*, *Cryptococcus*, *Mucor*, *Candida*, *Pneumocystis carinii*, and *Histoplasma capsulatum*.

### PCR amplification, result interpretation and quality control

The forward/reverse primers (10 μmol/L) and probes (10 μmol/L) in each reaction system were 0.4, 0.4, 0.4 μL, respectively. Two microliter template RNA (or positive and negative control) was added, and RNAase-free sterilized water was added to 10 μL for each reaction system. Fluorescence PCR amplification was performed as following parameters: 42°C for 5 min; 95°C for 10 s; 95°C for 5 s and 60°C for 34 s, these steps were repeated by 45 cycles and amplified by DX real-time quantitative PCR amplifier.

Positive results were defined as Ct value ≤40 and with an obvious amplification curve. If there is no amplification curve or the Ct value >40, it was considered as pathogen negative. We will run the test again if the amplification curve is atypical, if the result was still uncertain, and the sample collection was regarded as unqualified. RNP and GAPDH were used as internal references to estimate whether the samples were qualified in laboratory tests.

### Statistical analysis

Categorical variables were presented using frequencies, and continuous variables were summarized using median and interquartile ranges (IQR). The association between protective measures and respiratory pathogen colonization was assessed using age-adjusted and multivariable logistic regression models. We included age and sex into multivariable models a priori regardless of univariable results. Additional variables that were related (*p* < 0.1) to respiratory pathogen colonization in the univariable analysis were also included. Odds ratios (ORs) and 95% confidence intervals (CI) were calculated to describe the impact of differing protective measures on respiratory pathogen colonization. All statistical analyses were conducted using SPSS (version 23.0) and R statistical software (R Foundation for Statistical Computing).

## Results

### Characteristics of the study population

**Figure 1.**
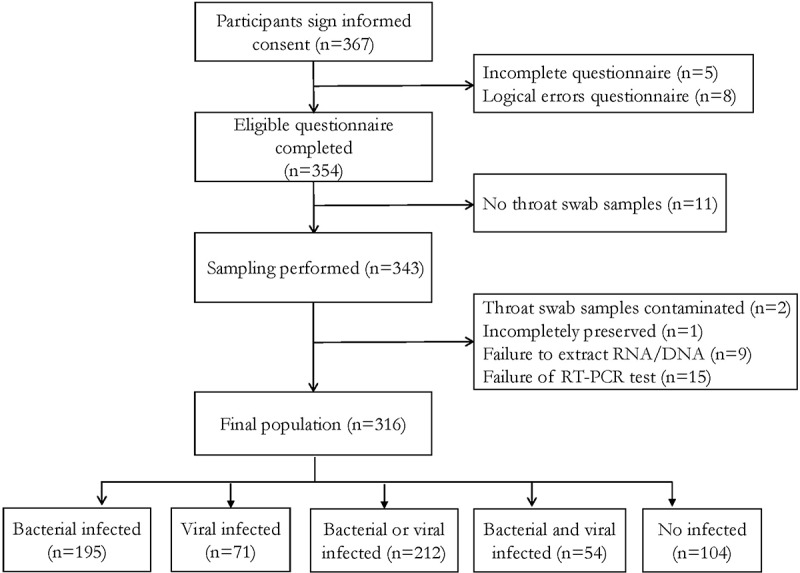
Flowchart of health care workers enrolled in the study from Jiangsu Province, China.

A total of 367 healthcare workers were enrolled from 2020 to 2021. Among them, 51 workers were excluded because of incomplete questionnaire (*n* = 5), logical errors questionnaire (*n* = 8), no throat swab samples (*n* = 11), throat swab samples contaminated (*n* = 2), incompletely preserved (*n* = 1), failure to extract RNA (*n* = 9), and failure of RT-PCR test (*n* = 15) (Figure 1). Of 316 healthcare workers, 143 and 173 participants were enrolled in 2020 and 2021, respectively. One hundred and eleven (35.1%) healthcare workers were males and 205 (64.9%) were females, and the median age was 36 (IQR, 29–43) years. Among them, 14 (4.4%) were underweight, 19 (6.0%) were obese, 13 (4.1%) had a tobacco smoking history, 72 (23.1%) had an alcohol drinking history, 96 (30.4%) had an influenza vaccination, and 303 (95.9%) had a COVID-19 vaccination (Table 1).

**Table 1.** Demographic characteristics of healthcare workers enrolled in the study.

Variable	Total	Bacterial infection	Virus infection	Bacterial or virus infection	Bacterial and virus infection
	N (%)	N (%)	N (%)	N (%)	N (%)
Male	111 (35.1)	75 (38.5)	34 (47.9)	86 (40.6)	23 (42.6)
Median Age (IQR)	36 (29–43)	36 (29–43)	36 (30–40)	36 (29–43)	36 (30–40)
Body Mass Index, kg/m2*					
<18.5	14 (4.4)	11 (5.6)	2 (2.8)	11 (5.2)	2 (3.7)
≥18.5 and < 24	209 (66.1)	132 (67.7)	46 (64.8)	138 (65.1)	40 (74.1)
≥24 and < 28	74 (23.4)	37 (19.0)	18 (25.4)	48 (22.6)	7 (13.0)
≥28	19 (6.0)	15 (7.7)	5 (7.0)	15 (7.1)	5 (9.3)
Enrolled year					
2021 year	173 (54.7)	100 (51.3)	28 (39.4)	108 (50.9)	20 (37.0)
2020 year	143 (45.3)	95 (48.7)	43 (60.6)	104 (49.1)	34 (63.0)
Degree of education					
Under high school	33 (10.4)	23 (11.8)	6 (8.5)	25 (11.8)	4 (7.4)
Bachelor’s degree	191 (60.4)	106 (54.4)	42 (59.2)	118 (55.7)	30 (55.6)
Above bachelor’s degree	92 (29.1)	66 (33.8)	23 (32.4)	69 (32.5)	20 (37.0)
Monthly income					
Less than 5000	19 (6.0)	13 (6.7)	5 (7.0)	14 (6.6)	4 (7.4)
5000–10000	137 (43.4)	79 (40.5)	26 (36.6)	86 (40.6)	19 (35.2)
10000–15000	127 (40.2)	80 (41.0)	29 (40.8)	86 (40.6)	23 (42.6)
More than 15,000	33 (10.4)	23 (11.8)	11 (15.5)	26 (12.3)	8 (14.8)
Smoking	13 (4.1)	10 (5.1)	5 (7.0)	11 (5.2)	4 (7.4)
Drinking	73 (23.1)	49 (25.1)	18 (25.4)	54 (25.5)	13 (24.1)
Influenza vaccination	96 (30.4)	59 (30.3)	20 (28.2)	63 (29.7)	16 (29.6)
COVID-19 vaccination	303 (95.9)	184 (94.4)	70 (98.6)	201 (94.8)	53 (98.1)
Respiratory disease of family members	16 (5.1)	12 (6.2)	6 (8.5)	14 (6.6)	4 (7.4)
Chronic Disease	26 (8.2)	20 (10.3)	6 (8.5)	20 (9.4)	6 (11.1)

Bacterial infection was present in 195 healthcare workers. Among them, 15 (7.7%) obesity, 10 (5.1%) had a tobacco smoking history, 49 (25.1%) had an alcohol drinking history. The percent of influenza vaccination and COVID-19 vaccination was 30.3% (59/195) and 94.4% (184/195), respectively. Their family members with respiratory diseases accounted for 6.2% (12/195). For virus infection, there were 71 healthcare workers (34 males and 37 females; median age 36, with the IQR of 30–40 years). There were 2 underweight, 46 normal weight, 18 overweight and 5 with obesity. Among participants, 5 (7.0%) had a tobacco smoking history and 18 (25.4%) had an alcohol drinking history. Two hundred and twelve healthcare workers were infected with either a bacterial or virus with a median age of 36 (IQR, 29–43) years. Fifty-four healthcare workers with both a bacterial and viral infection (23 males and 31 females) with a median age of 36 (IQR, 30–40) years (Table 1).

### Distribution of respiratory pathogens in health care workers

**Figure 2.**
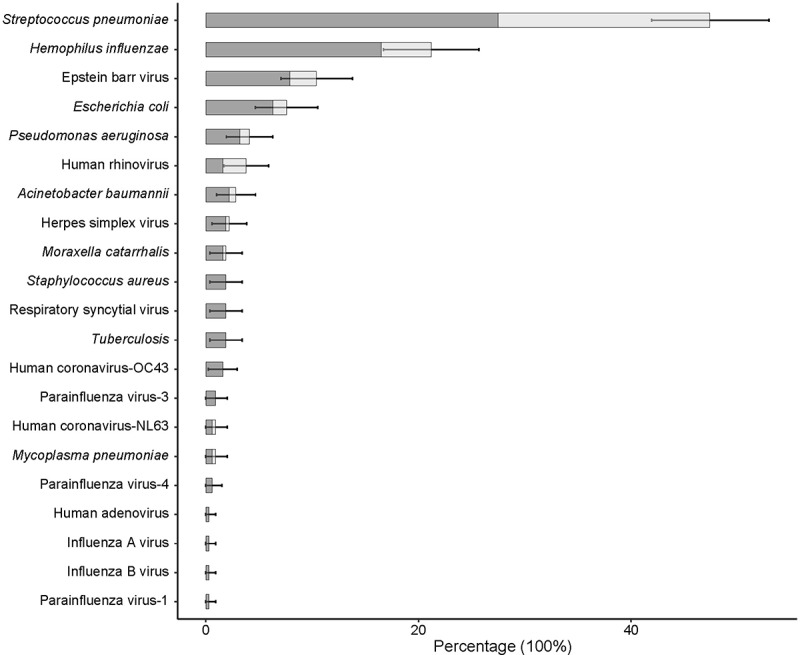
Distribution proportion of respiratory pathogens in health care workers.

In total, 67.1% of healthcare workers had a respiratory infection; colonization of respiratory bacteria and viruses were 61.7% and 22.5%, respectively. A total of 21 pathogens were detected (Figure 2). The most common pathogen was streptococcus pneumoniae (47.5%) followed by *Hemophilus influenzae* (21.2%), Epstein–Barr virus (10.4%), *Escherichia coli* (7.6%), *Pseudomonas aeruginosa* (4.1%), human rhinovirus (3.8%), *A. baumannii* (2.8%), and herpes simplex virus (2.2%), respectively. The prevalence of the remaining pathogens was less than 2%, ranging from 0.3% to 1.9%. When comparing the distribution of respiratory pathogens in both years of data collection, we found *Hemophilus influenzae* (29.37% vs 14.45%), human rhinovirus (6.30 vs 1.73), herpes simplex virus (4.90% vs 0.00) and human coronavirus-OC43 (3.50% vs 0.00) were higher in 2020 compared to 2021 (*p* < 0.05). The presence of any infection, any bacterial infection, any viral or bacterial infection were all higher in 2020 compared to 2021 (Supplementary Table S1, Supplementary Figure S1).

### Occupational factors of health care workers

Of 316 healthcare workers, 66 (20.9%) worked in the respiratory department, 138 (43.7%) were physicians, 133 (42.1%) were nurses, and 45 (14.2%) were medical technicians, administrative or labor personnel. Approximately 50% of participants worked more than 10 years and most worked more than 8 h per day. During daily work, 217 (68.7%) had contact with patients between 4 and 8 hours; 74 (23.4%) had contact with patients more than 8 h. Mask use was common; 98.1% of healthcare workers self-reported use of masks all the time. Of these, surgical mask use accounted for 86.1% of all mask use; disposable medical masks and N95 masks accounted for 12.0% and 1.9%, respectively. Approximately half of healthcare workers received IPC training ≤2 times per year, and 40.8% trained 3 or more times every year. As for 5 Moments for hand hygiene recommended by WHO, 63.3% fully understood details, and nearly all of them knew at least one point. However, only 69.0% fully implemented hand hygiene in daily work.

Among 71 healthcare workers were infected with a virus and the same as healthcare workers with bacterial infection, only about half of them fully implemented hand hygiene in daily work. About 28.2% healthcare workers infected with virus and 33.3% healthcare workers infected both virus and bacterial and never had IPC training, which were higher than other groups. Among a total of 54 healthcare workers infected with both bacterial and virus, 53.7% of them could not fully implement hand hygiene in daily work, which were higher than other groups (Table 2).

**Table 2.** Occupational factors of enrolled healthcare workers in this study.

Variable	Total	Bacterial infection	Virus infection	Bacterial or virus infection	Bacterial and virus infection
	N (%)	N (%)	N (%)	N (%)	N (%)
Occupation type					
Doctors	138 (43.7)	91 (46.7)	35 (49.3)	99 (46.7)	27 (50.0)
Nurse	133 (42.1)	71 (36.4)	27 (38.0)	78 (36.8)	20 (37.0)
Others	45 (14.2)	33 (16.9)	9 (12.7)	35 (16.5)	7 (13.0)
Works in the respiratory department	66 (20.9)	35 (17.9)	13 (18.3)	39 (18.4)	9 (16.7)
Occupation years					
<5 years	96 (30.4)	60 (30.8)	23 (32.4)	65 (30.7)	18 (33.3)
5–10 years	88 (27.8)	53 (27.2)	19 (26.8)	58 (27.4)	14 (25.9)
>10 years	132 (41.8)	82 (42.1)	29 (40.8)	89 (42.0)	22 (40.7)
Work time per day					
<8 hours	97 (30.7)	58 (29.7)	26 (36.6)	65 (30.7)	19 (35.2)
>8 hours	219 (69.3)	137 (70.3)	45 (63.4)	147 (69.3)	35 (64.8)
Contact with patients time per day					
<4 hours	25 (7.9)	15 (7.7)	5 (7.0)	15 (7.1)	5 (9.3)
4–8 hours	217 (68.7)	141 (72.3)	55 (77.5)	155 (73.1)	41 (75.9)
>8 hours	74 (23.4)	39 (20.0)	11 (15.5)	42 (19.8)	8 (14.8)
Self-reported mask adherence					
Good	310 (98.1)	189 (96.9)	66 (93.0)	206 (97.2)	49 (90.7)
Not good	6 (1.9)	6 (3.1)	5 (7.0)	6 (2.8)	5 (9.3)
Mask style					
N95	6 (1.9)	4 (2.1)	2 (2.8)	4 (1.9)	2 (3.7)
Surgical mask	272 (86.1)	164 (84.1)	58 (81.7)	178 (84.0)	44 (81.5)
Disposable medical mask	38 (12.0)	27 (13.8)	11 (15.5)	30 (14.2)	8 (14.8)
IPC training per year					
Never	27 (8.5)	24 (12.3)	20 (28.2)	26 (12.3)	18 (33.3)
Equal or less than 2 times	160 (50.6)	96 (49.2)	29 (40.8)	105 (49.5)	20 (37.0)
Equal or less than 3 times	129 (40.8)	75 (38.5)	22 (31.0)	81 (38.2)	16 (29.6)
Known of WHO 5 Moments for hand hygiene					
Part known	116 (36.7)	88 (45.1)	35 (49.3)	94 (44.3)	29 (53.7)
All known	200 (63.3)	107 (54.9)	36 (50.7)	118 (55.7)	25 (46.3)
WHO 5 Moments for hand hygiene					
Before touching a patient	299 (94.6)	185 (94.9)	69 (97.2)	201 (94.8)	53 (98.1)
Before clean/aseptic procedures	314 (99.4)	193 (99.0)	71 (100.0)	210 (99.1)	54 (100.0)
After body fluid exposure/risk	312 (98.7)	192 (98.5)	69 (97.2)	208 (98.1)	53 (98.1)
After touching a patient	316 (100.0)	195 (100.0)	71 (100.0)	212 (100.0)	54 (100.0)
After touching patient surroundings	303 (95.9)	186 (95.4)	67 (94.4)	202 (95.3)	51 (94.4)
Hand hygiene after glove removal	313 (99.1)	193 (99.0)	71 (100.0)	210 (99.1)	54 (100.0)
Hand hygiene implement					
Fully implement	218 (69.0)	114 (58.5)	36 (50.7)	125 (59.0)	25 (46.3)
Partly implement	98 (31.0)	81 (41.5)	35 (49.3)	87 (41.0)	29 (53.7)
Hand hygiene training					
Never	39 (12.3)	22 (11.3)	12 (16.9)	25 (11.8)	9 (16.7)
Equal or less than 2 times	175 (55.4)	113 (57.9)	38 (53.5)	123 (58.0)	28 (51.9)
Equal or less than 3 times	102 (32.3)	60 (30.8)	21 (29.6)	64 (30.2)	17 (31.5)
Air ventilation mode					
Natural ventilation	139 (44.0)	93 (47.7)	36 (50.7)	101 (47.6)	28 (51.9)
Mechanical ventilation	39 (12.3)	21 (10.8)	10 (14.1)	23 (10.8)	8 (14.8)
Central air conditioning ventilation	116 (36.7)	68 (34.9)	22 (31.0)	74 (34.9)	16 (29.6)
Air purifier	22 (7.0)	13 (6.7)	3 (4.2)	14 (6.6)	2 (3.7)
Air disinfection					
Ultraviolet ray	177 (56.0)	117 (60.0)	42 (59.1)	125 (59.0)	34 (63.0)
Recirculating air	77 (24.4)	46 (23.6)	22 (31.0)	53 (25.0)	15 (27.8)
Electrostatic adsorption	10 (3.2)	8 (4.1)	0 (0)	8 (3.8)	0 (0)
Chemical disinfectants	43 (13.6)	24 (12.3)	7 (9.9)	26 (12.3)	5 (9.3)

### Univariable analysis assessing characteristics associated with respiratory pathogens

We analyzed the effects of characteristics and occupational factors on microbial infection. For bacterial infection, healthcare workers with overweight, working as nurses, wearing masks all the time, accepting IPC training, fully understanding WHO 5 Moments for hand hygiene, and fully implement of hand hygiene helped to reduce infecting bacterial. As for virus infection, sex, enrolled year, whether to participate in IPC training, known of WHO 5 Moments for hand hygiene, and implementation of hand hygiene were significantly associated with the infection of viruses. In regard of healthcare workers infected with either bacterial or virus, male, enrolled in 2020, working as doctors, never accepting IPC training, partly known of WHO 5 Moments for hand hygiene, and partly implementing hand hygiene were considered as risk factors of the infection. In addition, overweight, enrolled in 2021, good self-reported mask adherence, attending IPC training, fully known of WHO 5 Moments for hand hygiene, and fully implementing hand hygiene decreased infected risk of both bacterial and virus (Supplementary Table S2).

### Multivariable assessing characteristics associated with respiratory pathogens

**Figure 3.**
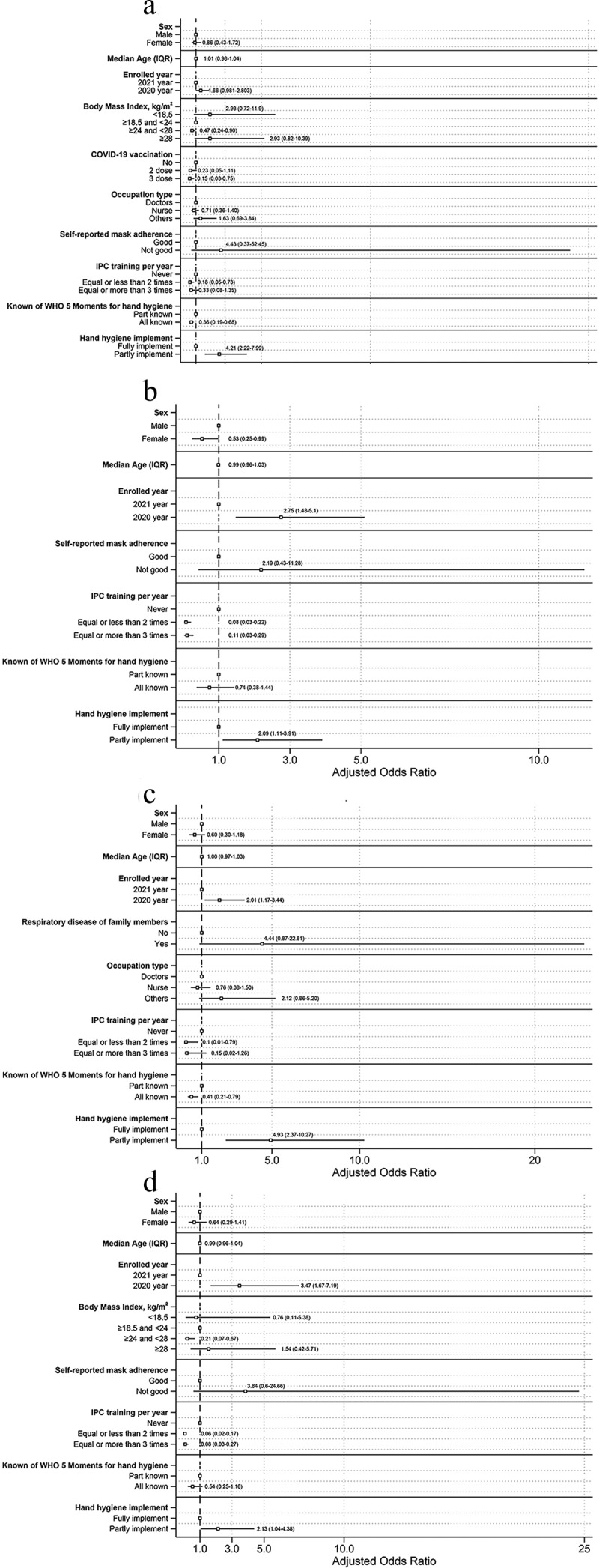
Multivariable analysis in health care workers assessing characteristics associated with respiratory pathogens.

We further performed a multivariable analysis to assess characteristics associated with microbial infection by considering sex, age and factors based on univariable analysis (*p* < 0.1) (Figure 3). For bacterial infection, enrolled year, body mass index (BMI), COVID-19 vaccination, Self-reported mask adherence, IPC training per year, occupation type, known of WHO 5 Moments for hand hygiene, hand hygiene implement were finally left in the model, suggesting that overweight (adjusted odds ratio [aOR], 0.47; 95% CI, 0.24–0.90; *p* = 0.024), 3 dose COVID-19 vaccination (aOR, 0.15; 95% CI: 0.03–0.75; *p* = 0.021), accepting IPC training equal or less than 2 times per year (aOR, 0.18; 95% CI, 0.05–0.73;*p* = 0.016), all known of WHO 5 Moments for hand hygiene (aOR, 0.36; 95% CI, 0.19–0.68; *p* = 0.002), and partly implementing hand hygiene (aOR, 4.21; 95% CI: 2.22–7.99; *p* < 0.001) were significantly associated with bacterial infection. For virus infection, results showed female (aOR, 0.53; 95% CI, 0.28–0.99; *p* = 0.048), participants enrolled in 2020 (aOR: 2.75, 95% CI: 1.48–5.10, *p* = 0.001), participating in IPC training equal or less than 2 times per year (aOR: 0.08, 95% CI: 0.03–0.22, *p* < 0.001), equal or more than 3 times per year (aOR, 0.11; 95% CI, 0.03–0.29; *p* < 0.001), and partly implementing hand hygiene (aOR, 2.09; 95% CI, 1.11–3.91; *p* = 0.022) were strongly related to virus infection. For healthcare workers infected with bacterial or virus infection, results showed enrolled in 2020 (aOR, 2.01; 95% CI; 1.17–3.44; *p* = 0.011), participating in IPC training equal or less than 2 times per year (aOR, 0.10; 95% CI, 0.01–0.79; *p* = 0.029), all known of WHO 5 Moments for hand hygiene (aOR, 0.41; 95% CI, 0.21–0.79; *p* = 0.007), and partly implementing hand hygiene (aOR, 4.93, 95% CI;, 2.37–10.27; *p* < 0.001) were related to bacterial or virus infection. In terms of both bacterial and virus infection, enrolled year (OR, 3.47; 95% CI, 1.67–7.19; *p* = 0.001), overweight (aOR, 0.21; 95% CI, 0.07–0.67; *p* = 0.008), IPC training ≥2 times per year (aOR, 0.06; 95% CI, 0.02–0.17; *p* < 0.001; aOR: 0.08, 95% CI: 0.03–0.27, *p* < 0.001), and partly implementing hand hygiene (aOR, 2.13; 95% CI, 1.04–4.38; *p* = 0.040) were factors to infected with both bacterial and virus.

## Discussion

Occupational exposures of healthcare workers are important, frequently overlooked, and modifiable contributors to the burden of respiratory disease [15]. The COVID-19 pandemic challenges healthcare workers' safety in part because of limited supplies of PPE [16]. Quantifying the occupational contribution to this disease burden is critical to preventing disease and improving lung health. To date, the question of the occupational burden in respiratory disease of healthcare workers in Jiangsu province has not been well known, especially during the COVID-19 pandemic. Our results showed that high respiratory infection among healthcare workers and occupational factors relate to respiratory infection suggest that we should pay more attention to healthcare workers’ IPC training and PPE.

In this study, we found respiratory infections more common in 2020 than in 2021. In Early 2020, this lack of awareness, coupled with limited information accessibility, meant frontline HCWs were not taking adequate protective measures [17,18]. Shortages of adequate PPE are a constant worry in 2020 [9]. Compounding this risk, the COVID-19 surge has led to increased patient loads and longer working hours for HCWs. This exhaustion suppresses their immune systems, making them even more susceptible to contracting pathogens [8,19]. Beyond analyzing variations in HCW infection rates throughout the pandemic, future studies can examine pre- and post-pandemic infection rates to inform the development of more effective healthcare worker protection policies.

Respiratory infection has the highest incidence rate among all nosocomial infections and is the focus of nosocomial infection prevention and control [20]. Several studies have investigated bacterial and viral colonization among healthcare workers. A prospective cohort study in China found that a significant portion (88%) of participants harbored bacterial colonization at the beginning or end of the study. Of these, over two-thirds (66%) had only bacteria, while the remaining participants (22%) had a co-infection with a virus [6]. Previous observational studies during different seasons paint a similar picture. Studies report that 34–37% of healthcare workers carry at least one type of bacteria, with viral co-infections ranging from 4% to 11% [7]. Research from Chicago identified a positive respiratory pathogen test result in 243 healthcare workers, with 243 (54%) HCWs had a positive test for any respiratory pathogen. Our study found that a significant majority (67.1% or 212 participants) of healthcare workers tested positive for at least one respiratory pathogen. Bacterial pathogens were identified in 61.7% (195 participants), while 22.2% (70 participants) had a viral infection. These findings align with the results reported by Raina MacIntyre et al. [6]. However, some discrepancies exist when compared to studies from other regions. These variations might be attributed to differences in the studied populations and regions, which can also influence factors like patient pathogen load, exposure extent, and testing methodologies [10].

In our study, 67.1% of healthcare workers had a respiratory infection; colonization of respiratory bacteria and viruses were 61.7% and 22.5%, respectively. Streptococcus pneumoniae was the most prevalent pathogen (47.5%), followed by hemophilus influenzae (21.2%). This aligns with research by Hosuru Subramanya et al., who found that 65% of healthcare workers were colonized with *S. pneumoniae* and/or *Hemophilus influenzae*, compared to only 32% of non-healthcare workers [21]. In particular, the presence of *Streptococcus pneumoniae*, *Haemophilus influenzae* and Epstein Barr virus were distinct from previously published studies in Chinese [22,23]. Some of these pathogens (*Streptococcus pneumoniae*, *Haemophilus influenzae*, *Escherichia coli*, respiratory syncytial virus, etc.) infection rates higher than other community-acquired pneumonia study [24].

Previous studies suggest that some respiratory viruses (e.g. influenza, severe acute respiratory syndrome coronavirus, rhinovirus) that are transmitted primarily by droplets and/or contact might simultaneously be spread through aerosol under certain conditions and perhaps by certain patients [25,26]. Occupational risks in the workplace must be minimized if not altogether eliminated. It is essential that measures are put in place to ensure that healthcare workers are continually protected [27]. Respiratory protection is a key strategy for pandemic control and key to sustaining the healthcare workforce. Before COVID-19 in China, the need for healthcare workers to wear masks during non-high-risk operations was highly controversial, but now masks have become mandatory since COVID-19. In this study, we found that the infection rate of some respiratory pathogens in healthcare workers was higher in the initial outbreak of COVID-19 than in the regular epidemic prevention and control period. Among the 316 healthcare workers included in this study, 98.1% of them self-reported mask adherence were good, and only 1.9% healthcare workers said that they sometimes forgot to wear mask. There was no significant difference in the detection rate of respiratory infection among different masks. MacIntyre showed that there were no significant differences between N95 respirators and medical masks for the four primary outcomes in the adjusted analysis [28]. What we found in our study that was closely related to respiratory pathogen infection was good adherence and replacement frequency which was found similar in other studies [29,30].

Studies have shown that the hands of medical staff carry a variety of pathogenic bacteria, and good hand hygiene habits can effectively reduce the damage to various respiratory pathogens [31]. Similarly, due to the impact of COVID-19 epidemic and IPC training in hospitals, hand hygiene compliance of medical staff has been greatly improved [32]. Approximately 30% of the medical staff still said that they were not able to fully implement hand hygiene as a part of their daily work. Reasons for this included being too busy, detergent and disinfectant skin irritation caused by skin dryness, and individuals not paying attention to hand washing during work. A multivariable analysis demonstrated that incomplete implementation of hand hygiene as part of daily work was a risk factor for respiratory pathogen colonization in healthcare workers. Knowledge of WHO critieria for hand hygiene was a protective factor for respiratory bacterial infection and respiratory bacterial or virus infection.

IPC training is an effective measure to reduce various occupational infection [33]. In our study, 91.4% of healthcare workers participated in IPC training more than 1 time within 1 year, and 40.8% of them participated more than three times. The detection rate of respiratory bacteria and viruses among healthcare workers who had participated in IPC training was relatively low, and the difference was statistically significant (*p* < 0.05). Hospitals should provide adequate IPC and PPE supplies during COVID-19 epidemic [34]. In our study, the vast majority healthcare workers believed that it was necessary to conduct regular IPC training. IPC training contents include the concept of occupational exposure, the content of the standard prevention, risk factors and protection skills, processing flow after occupational exposure, etc. [35].

This study has several limitations. First, the survey was carried out and administered several weeks after possible exposures, and therefore recall bias is a possibility. Recall bias would limit assessment of important variables, such as frequency of exposure and duration of contact during specific procedures. Second, only two general hospitals were included in this study, and the generalizability of these findings outside China is unclear. These findings should be validated in settings outside of this province. Finally, this study did not collect respiratory pathogen samples from healthcare workers at distinct time points. Many of these pathogens may be transient and nonconsequential. Serial sampling in the same individuals would be useful for understanding the course of infection and clinical disease of each of these pathogens.

## Conclusions

In conclusion, 67.1% healthcare workers had at least one respiratory pathogen colonization. Colonization of bacteria was much more common than viruses, and overall rates were higher in 2020 compared to 2021. Different PPE and IPC measures were associated with viral and/or bacterial colonization.

## List of abbreviations

PPE: Personal Protective Equipment
IPC: Infection Prevention and Control
IQR: Interquartile Ranges
ORs: Odds Ratios
CI: Confidence Intervals
BMI: Body Mass Index
aOR: Adjusted Odds Ratio

## Supplementary Material

Supplementary.docx

## Data Availability

Please contact the first author for data requests.
