# Supplementary material for: Colonization of bacterial and viral respiratory pathogens among healthcare workers in China during COVID–19 pandemic
Source: J Oral Microbiol. 2024 Jun 20;16(1):2365965. doi: 10.1080/20002297.2024.2365965 (PMC11191836; doi:10.1080/20002297.2024.2365965)
Supplement: Supplementary.docx [file ZJOM_A_2365965_SM0264.docx]

**Supplementary Tables and Figures.**

Colonization of bacterial and viral respiratory pathogens among healthcare workers in Eastern China during COVID–19 pandemic.

Supplementary Table 1. Comparison of the characteristics between respiratory pathogens by different years

Supplementary Table 2. Univariable analysis assessing characteristics associated with respiratory pathogens.

Supplementary Figure 1. Distribution proportion of respiratory pathogens by different years

Supplementary file. Medical personnel safety and protection survey questionnaire

Supplementary Table 1. Comparison of the characteristics between respiratory pathogens by different years

| Respiratory pathogens | Total | | 2020 | | 2021 | | *P-*value |
| --- | --- | --- | --- | --- | --- | --- | --- |
|  | N | % | N | % | N | % |  |
| Streptococcus pneumoniae | 150 | 47.47 | 70 | 48.95 | 80 | 46.24 | 0.631 |
| Hemophilus influenzae | 67 | 21.20 | 42 | 29.37 | 25 | 14.45 | 0.001 |
| Epstein barr virus | 33 | 10.44 | 10 | 6.99 | 23 | 13.29 | 0.068 |
| Escherichia coli | 24 | 7.59 | 11 | 7.69 | 13 | 7.51 | 0.953 |
| Pseudomonas aeruginosa | 13 | 4.11 | 5 | 3.50 | 8 | 4.62 | 0.615 |
| Human rhinovirus | 12 | 3.80 | 9 | 6.30 | 3 | 1.73 | 0.035 |
| Acinetobacter baumannii | 9 | 2.85 | 4 | 2.80 | 5 | 2.89 | 1* |
| Herpes simplex virus | 7 | 2.22 | 7 | 4.90 | 0 | 0.00 | 0.004* |
| Moraxella catarrhalis | 6 | 1.90 | 4 | 2.80 | 2 | 1.16 | 0.416* |
| Staphylococcus aureus | 6 | 1.90 | 4 | 2.80 | 2 | 1.16 | 0.416* |
| Respiratory syncytial virus | 6 | 1.90 | 5 | 3.50 | 1 | 0.58 | 0.095* |
| Tuberculosis | 6 | 1.90 | 0 | 0.00 | 6 | 3.47 | 0.034* |
| Human coronavirus−OC43 | 5 | 1.58 | 5 | 3.50 | 0 | 0.00 | 0.018* |
| Parainfluenza virus-3 | 3 | 0.95 | 2 | 1.40 | 1 | 0.58 | 0.592* |
| Human coronavirus−NL63 | 3 | 0.95 | 3 | 2.10 | 0 | 0.00 | 0.092* |
| Mycoplasma pneumoniae | 3 | 0.95 | 0 | 0.00 | 3 | 1.73 | 0.254* |
| Parainfluenza virus-4 | 2 | 0.63 | 2 | 1.40 | 0 | 0.00 | 0.204* |
| Human adenovirus | 1 | 0.32 | 1 | 0.70 | 0 | 0.00 | 0.453* |
| Influenza A virus | 1 | 0.32 | 1 | 0.70 | 0 | 0.00 | 0.453* |
| Influenza B virus | 1 | 0.32 | 1 | 0.70 | 0 | 0.00 | 0.453* |
| Parainfluenza virus-1 | 1 | 0.32 | 1 | 0.70 | 0 | 0.00 | 0.453* |
| Bordetella pertussis | 0 | 0.00 | 0 | 0.00 | 0 | 0.00 | N/A |
| Chlamydia pneumoniae | 0 | 0.00 | 0 | 0.00 | 0 | 0.00 | N/A |
| Legionella pneumophila | 0 | 0.00 | 0 | 0.00 | 0 | 0.00 | N/A |
| Klebsiella pneumoniae | 0 | 0.00 | 0 | 0.00 | 0 | 0.00 | N/A |
| Cytomegalovirus | 0 | 0.00 | 0 | 0.00 | 0 | 0.00 | N/A |
| Parainfluenza virus-2 | 0 | 0.00 | 0 | 0.00 | 0 | 0.00 | N/A |
| Human coronavirus−HKU1 | 0 | 0.00 | 0 | 0.00 | 0 | 0.00 | N/A |
| Human coronavirus−229E | 0 | 0.00 | 0 | 0.00 | 0 | 0.00 | N/A |
| Any virus | 71 | 22.47 | 43 | 30.07 | 28 | 16.18 | 0.003 |
| Any bacteria | 195 | 61.71 | 95 | 66.43 | 100 | 57.80 | 0.116 |
| Any virus or bacteria | 212 | 67.09 | 104 | 72.73 | 108 | 62.43 | 0.052 |
| Any virus and bacteria | 54 | 17.09 | 34 | 23.78 | 20 | 11.56 | 0.004 |

* Fisher's Exact Test

Supplementary Table 2. Univariable analysis assessing characteristics associated with respiratory pathogens.

| Variable | Bacterial infection | | Virus infection | | Bacterial or virus infection | | Bacterial and virus infection | |
| --- | --- | --- | --- | --- | --- | --- | --- | --- |
|  | N (%) | Odds Ratio (95% CI), *P* | N (%) | Odds Ratio (95% CI), *P* | N (%) | Odds Ratio (95% CI), *P* | N (%) | Odds Ratio (95% CI), *P* |
| Sex |  |  |  |  |  |  |  |  |
| Male | 75 (38.5) | Referent | 34 (47.9) | Referent | 86 (40.6) | Referent | 23 (42.6) | Referent |
| Female | 120 (61.5) | 0.68 (0.42-1.10), 0.116 | 37 (52.1) | 0.50 (0.29-0.85), 0.011 | 126 (59.4) | 0.46 (0.27-0.79), 0.004 | 31 (57.4) | 0.68 (0.38-1.24), 0.209 |
| Median Age (IQR) | 36 (29-43) | 1.00 (0.98-1.03), 0.742 | 36 (30-40) | 0.99 (0.96-1.02), 0.665 | 36 (29-43) | 1.01 (0.98-1.03), 0.686 | 36 (30-40) | 0.99 (0.96-1.03), 0.577 |
| Body Mass Index, kg/m2* |  |  |  |  |  |  |  |  |
| <18.5 | 11 (5.6) | 2.14 (0.58-7.91), 0.254 | 2 (2.8) | 0.59 (0.13-2.73), 0.501 | 11 (5.2) | 1.89 (0.51-6.98), 0.342 | 2 (3.7） | 0.70 (0.15-3.72), 0.65 |
| ≥18.5 and <24 | 132 (67.7) | Referent | 46 (64.8) | Referent | 138 (65.1) | Referent | 40 (74.1) | Referent |
| ≥24 and <28 | 37 (19.0) | 0.58 (0.34-0.99), 0.048 | 18 (25.4) | 1.14 (0.61-2.13), 0.683 | 48 (22.6) | 0.95 (0.54-1.66), 0.863 | 7 (13.0) | 0.44 (0.19-1.03), 0.060 |
| ≥28 | 15 (7.7) | 2.19 (0.70-6.83), 0.178 | 5 (7.0) | 1.27 (0.43-3.70), 0.667 | 15 (7.1) | 1.93 (0.62-6.03), 0.258 | 5 (9.3) | 1.51 (0.51-4.43), 0.454 |
| Enrolled year |  |  |  |  |  |  |  |  |
| 2021 year | 100 (51.3) | Referent | 28 (39.4) | Referent | 108 (50.9) | Referent | 20 (37.0) | Referent |
| 2020 year | 95 (48.7) | 1.45 (0.91–2.29), 0.117 | 43 (60.6) | 2.23 (1.30–3.82), 0.004 | 104 (49.1) | 1.61 (0.99–2.59), 0.053 | 34 (63.0) | 2.39 (1.30–4.37), 0.005 |
| Degree of education |  |  |  |  |  |  |  |  |
| Under high school | 23 (11.8) | Referent | 6 (8.5) | Referent | 25 (11.8) | Referent | 4 (7.4) | Referent |
| Bachelor degree | 106 (54.4) | 0.54 (0.25-1.20), 0.131 | 42 (59.2) | 1.27 (0.49-3.28), 0.623 | 118 (55.7) | 0.52 (0.22-1.21), 0.128 | 30 (55.6) | 1.35 (0.44-4.12), 0.597 |
| Above bachelor degree | 66 (33.8) | 1.10 (0.46-2.64), 0.824 | 23 (32.4) | 1.50 (0.55-4.09), 0.428 | 69 (32.5) | 0.96 (0.38-2.42), 0.931 | 20 (37.0) | 2.01 (0.63-6.40), 0.236 |
| Monthly income |  |  |  |  |  |  |  |  |
| Less than 5000 | 13 (6.7) | Referent | 5 (7.0) | Referent | 14 (6.6) | Referent | 4 (7.4) | Referent |
| 5000-10000 | 79 (40.5) | 0.63 (0.23-1.75), 0.375 | 26 (36.6) | 0.66 (0.22-1.98), 0.455 | 86 (40.6) | 0.60 (0.21-1.77), 0.357 | 19 (35.2) | 0.60 (0.18-2.01), 0.412 |
| 10000-15000 | 80 (41.0) | 0.79 (0.28-2.21), 0.647 | 29 (40.8) | 0.83 (0.28-2.49), 0.738 | 86 (40.6) | 0.75 (0.25-2.22), 0.602 | 23 (42.6) | 0.83 (0.25-2.73), 0.758 |
| More than 15000 | 23 (11.8) | 1.06 (0.31-3.59), 0.924 | 11 (15.5) | 1.40 (0.40-4.89), 0.598 | 26 (12.3) | 1.33 (0.36-4.96), 0.675 | 8 (14.8) | 1.20 (0.31-4.68), 0.790 |
| Smoking |  |  |  |  |  |  |  |  |
| No | 185 (94.9) | Referent | 66 (93.0) | Referent | 201 (94.8) | Referent | 50 (92.6) | Referent |
| Yes | 10 (5.1) | 2.13 (0.57-7.89), 0.259 | 5 (7.0) | 2.24 (0.71-7.09), 0.168 | 11 (5.2) | 2.79 (0.61-12.83), 0.187 | 4 (7.4) | 2.25 (0.67-7.59), 0.192 |
| Drinking |  |  |  |  |  |  |  |  |
| No | 146 (74.9) | Referent | 53 (74.6) | Referent | 158 (74.5) | Referent | 41 (75.9) | Referent |
| Yes | 49 (25.1) | 1.36 (0.78-2.34), 0.279 | 18 (25.4) | 1.17 (0.64-2.17), 0.610 | 54 (25.5) | 1.53 (0.85-2.75), 0.155 | 13 (24.1) | 1.07 (0.54-2.12), 0.852 |
| Influenza vaccination |  |  |  |  |  |  |  |  |
| No | 136 (69.7) | Referent | 51 (71.8) | Referent | 149 (70.3) | Referent | 38 (70.4) | Referent |
| Yes | 59 (30.3) | 1.02 (0.62-1.66), 0.952 | 20 (28.2) | 1.15 (0.64-2.06), 0.646 | 63 (29.7) | 1.10 (0.66-1.83), 0.715 | 16 (29.6) | 1.04 (0.55-1.98), 0.895 |
| COVID-19 vaccination |  |  |  |  |  |  |  |  |
| No | 11 (5.6) | Referent | 1 (1.4) | Referent | 11 (5.2) | Referent | 1 (1.9) | Referent |
| 2 dose | 104 (53.3) | 0.32 (0.07-1.50), 0.148 | 41 (57.7) | 4.03 (0.51-31.97), 0.187 | 113 (53.3) | 0.41 (0.09-1.92), 0.259 | 32 (59.3) | 2.91 (0.37-23.38), 0.310 |
| 3 dose | 80 (41.0) | 0.24 (0.05-1.14), 0.072 | 29 (40.8) | 3.14 (0.39-25.11), 0.282 | 88 (41.5) | 0.31 (0.07-1.44), 0.135 | 21 (38.9) | 2.12 (0.26-17.16), 0.482 |
| Respiratory disease of family members | |  |  |  |  |  |  |  |
| No | 183 (93.8) | Referent | 65 (91.5) | Referent | 198 (93.4) | Referent | 50 (92.6) | Referent |
| Yes | 12 (6.2) | 1.92 (0.60-6.09), 0.269 | 6 (8.5) | 2.17 (0.76-6.19), 0.148 | 14 (6.6) | 3.61 (0.80-16.17), 0.094 | 4 (7.4) | 1.67 (0.52-5.38), 0.393 |
| Chronic Disease |  |  |  |  |  |  |  |  |
| No | 175 (89.7) | Referent | 65 (91.5) | Referent | 192 (90.6) | Referent | 48 (88.9) | Referent |
| Yes | 20 (10.3) | 2.19 (0.85-5.62), 0.103 | 6 (8.5) | 1.04 (0.40-2.69), 0.938 | 20 (9.4) | 1.70 (0.66-4.37), 0.270 | 6 (11.1) | 1.51 (0.58-3.96), 0.400 |
| Works in the respiratory department | |  |  |  |  |  |  |  |
| No | 160 (82.1) | Referent | 58 (81.7) | Referent | 173 (81.6) | Referent | 45 (83.3) | Referent |
| Yes | 35 (17.9) | 0.64 (0.37-1.10), 0.104 | 13 (18.3) | 0.81 (0.41-1.59), 0.545 | 39 (18.4) | 0.64 (0.37-1.13), 0.122 | 9 (16.7) | 0.72 (0.33-1.56), 0.404 |
| Occupation type |  |  |  |  |  |  |  |  |
| Doctors | 91 (46.7) | Referent | 35 (49.3) | Referent | 99 (46.7) | Referent | 27 (50.0) | Referent |
| Nurse | 71 (36.4) | 0.59 (0.36-0.97), 0.036 | 27 (38.0) | 0.75 (0.42-1.33), 0.322 | 78 (36.8) | 0.56 (0.34-0.93), 0.024 | 20 (37.0) | 0.73 (0.39-1.37), 0.326 |
| Others | 33 (16.9) | 1.42 (0.67-3.00), 0.358 | 9 (12.7) | 0.74 (0.32-1.68), 0.466 | 35 (16.5) | 1.38 (0.62-3.05), 0.428 | 7 (13.0) | 0.76 (0.31-1.88), 0.549 |
| Occupation years |  |  |  |  |  |  |  |  |
| <5 years | 60 (30.8) | Referent | 23 (32.4) | Referent | 65 (30.7) | Referent | 18 (33.3) | Referent |
| 5-10 years | 53 (27.2) | 0.91 (0.50-1.65), 0.752 | 19 (26.8) | 0.87 (0.44-1.74), 0.702 | 58 (27.4) | 0.92 (0.50-1.70), 0796 | 14 (25.9) | 0.82 (0.38-1.77), 0.612 |
| > 10 years | 82 (42.1) | 0.98 (0.57-1.69), 0.954 | 29 (40.8) | 0.89 (0.48-1.67), 0.724 | 89 (42.0) | 0.99 (0.56-1.73), 0.964 | 22 (40.7) | 0.87 (0.44-1.72), 0.683 |
| Work time per day |  |  |  |  |  |  |  |  |
| Less than 8 hours | 58 (29.7) | Referent | 26 (36.6) | Referent | 65 (30.7) | Referent | 19 (35.2) | Referent |
| Equal or more than 8 hours | 137 (70.3) | 1.12 (0.69-1.83), 0.641 | 45 (63.4) | 0.71 (0.41-1.23), 0.220 | 147 (69.3) | 1.00 (0.60-1.67), 0.984 | 35 (64.8) | 0.78 (0.42-1.45), 0.433 |
| Contact with patients time per day | |  |  |  |  |  |  |  |
| < 4 hours | 15 (7.7) | Referent | 5 (7.0) | Referent | 15 (7.1) | Referent | 5 (9.3) | Referent |
| 4-8 hours | 141 (72.3) | 1.24 (0.53-2.89), 0.623 | 55 (77.5) | 1.36 (0.49-3.79), 0.560 | 155 (73.1) | 1.67 (0.71-3.91), 0.240 | 41 (75.9) | 0.93 (0.33-2.63), 0.894 |
| > 8 hours | 39 (20.0) | 0.74 (0.30-1.87), 0.527 | 11 (15.5) | 0.70 (0.22-2.25), 0.551 | 42 (19.8) | 0.88 (0.35-2.20), 0.781 | 8 (14.8) | 0.49 (0.14-1.65), 0.246 |
| Self-reported mask adherence |  |  |  |  |  |  |  |  |
| Good | 189 (96.9) | Referent | 66 (93.0) | Referent | 206 (97.2) | Referent | 49 (90.7) | Referent |
| Not good | 6 (3.1) | 5.56 (1.52-20.29), 0.009 | 5 (7.0) | 5.81 (0.73-46.42), 0.097 | 6 (2.8) | 4.57 (0.57-36.54), 0.152 | 5 (9.3) | 8.06 (2.19-29.65), 0.002 |
| Mask style |  |  |  |  |  |  |  |  |
| N95 | 4 (2.1) | Referent | 2 (2.8) | Referent | 4 (1.9) | Referent | 2 (3.7) | Referent |
| Surgical mask | 164 (84.1) | 0.76 (0.14-4.22), 0.753 | 58 (81.7) | 0.54 (0.10-3.03), 0.486 | 178 (84.0) | 0.95 (0.17-5.27), 0.950 | 44 (81.5) | 0.39 (0.07-2.17), 0.280 |
| Disposable medical mask | 27 (13.8) | 1.23 (0.20-7.70), 0.827 | 11 (15.5) | 0.82 (0.13-5.11), 0.827 | 30 (14.2) | 1.88 (0.29-12.14), 0.510 | 8 (14.8) | 0.53 (0.08-3.45), 0.510 |
| IPC training per year | |  |  |  |  |  |  |  |
| Never | 24 (12.3) | Referent | 20 (28.2) | Referent | 26 (12.3) | Referent | 18 (33.3) | Referent |
| Equal or less than 2 times | 96 (49.2) | 0.19 (0.05-0.65), 0.008 | 29 (40.8) | 0.08 (0.03-0.20), <0.001 | 105 (49.5) | 0.07 (0.01-0.56), 0.11 | 20 (37.0) | 0.07 (0.03-0.18), <0.001 |
| Equal or more than 3 times | 75 (38.5) | 0.17 (0.05-0.61), 0.006 | 22 (31.0) | 0.07 (0.03-0.19), 0.072 | 81 (38.2) | 0.07 (0.01-0.49), 0.008 | 16 (29.6) | 0.07 (0.03-0.18), <0.001 |
| Known of WHO 5 Moments for hand hygiene | |  |  |  |  |  |  |  |
| Part known | 88 (45.1) | Referent | 35 (49.3) | Referent | 94 (44.3) | Referent | 29 (53.7) | Referent |
| All known | 107 (54.9) | 0.37 (0.22-0.61), <0.001 | 36 (50.7) | 0.51 (0.30-0.87), 0.013 | 118 (55.7) | 0.34 (0.20-0.58), <0.001 | 25 (46.3) | 0.43 (0.24-0.78), 0.005 |
| Hand hygiene before touching a patient | |  |  |  |  |  |  |  |
| Yes | 185 (94.9) | Referent | 69 (97.2) | Referent | 201 (94.8) | Referent | 53 (98.1) | Referent |
| No | 10 (5.1) | 0.88 (0.33-2.38), 0.801 | 2 (2.8) | 0.44 (0.10-1.99), 0.289 | 11 (5.2) | 0.89 (0.32-2.49), 0.830 | 1 (1.9) | 0.29 (0.04-2.34), 0.235 |
| Hand hygiene after touching patient surroundings | | |  |  |  |  |  |  |
| Yes | 186 (95.4) | Referent | 67 (94.4) | Referent | 202 (95.3) | Referent | 51 (94.4) | Referent |
| No | 9 (4.6) | 1.42 (0.43-4.70), 0.571 | 4 (5.6) | 1.57 (0.47-5.24), 0.472 | 10 (4.7) | 1.67 (0.45-6.19), 0.445 | 3 (5.6) | 1.48 (0.39-5.58), 0.560 |
| Hand hygiene implement |  |  |  |  |  |  |  |  |
| Fully implement | 114 (58.5) | Referent | 36 (50.7) | Referent | 125 (59.0) | Referent | 25 (46.3) | Referent |
| Partly implement | 81 (41.5) | 4.35 (2.42-7.81), <0.001 | 35 (49.3) | 2.81 (1.63-4.85), <0.001 | 87 (41.0) | 5.88 (2.98-11.64), <0.001 | 29 (53.7) | 3.25 (1.78-5.92), <0.001 |
| Hand hygiene training |  |  |  |  |  |  |  |  |
| Never | 22 (11.3) | Referent | 12 (16.9) | Referent | 25 (11.8) | Referent | 9 (16.7) | Referent |
| Equal or less than 2 times | 113 (57.9) | 1.41 (0.70-2.85), 0.341 | 38 (53.5) | 0.62 (0.29-1.35), 0.230 | 123 (58.0) | 1.33 (0.64-2.75), 0.450 | 28 (51.9) | 0.64 (0.27-1.48), 0.293 |
| Equal or more than 3 times | 60 (30.8) | 1.10 (0.52-2.33), 0.795 | 21 (29.6) | 0.58 (0.25-1.34), 0.204 | 64 (30.2) | 0.94 (0.44-2.03), 0.881 | 17 (31.5) | 0.67 (0.27-1.65), 0.382 |
| Air ventilation mode |  |  |  |  |  |  |  |  |
| Natural ventilation | 93 (47.7) | Referent | 36 (50.7) | Referent | 101 (47.6) | Referent | 28 (51.9) | Referent |
| Mechanical ventilation | 21 (10.8) | 0.58 (0.28-1.19), 0.136 | 10 (14.1) | 0.99 (0.44-2.22), 0.974 | 23 (10.8) | 0.54 (0.26-1.13), 0.103 | 8 (14.8) | 1.02 (0.42-2.47), 0.960 |
| Central air conditioning ventilation | 68 (34.9) | 0.70 (0.42-1.17), 0.173 | 22 (31.0) | 0.67 (0.37-1.22), 0.190 | 74 (34.9) | 0.66 (0.39-1.13), 0.129 | 16 (29.6) | 0.63 (0.32-1.24), 0.184 |
| Air purifier | 13 (6.7) | 0.71 (0.29-1.79), 0.714 | 3 (4.2) | 0.45 (0.13-1.62), 0.222 | 14 (6.6) | 0.66 (0.26-1.70), 0.386 | 2 (3.7) | 0.40 (0.09-1.80), 0.230 |

Supplementary Figure 1. Distribution proportion of respiratory pathogens by different years

Supplementary file. Medical personnel safety and protection survey questionnaire

Work unit _______________ Department _______________ Name：

**General demographic characteristics**

1. Sex： A. Male B. Female
2. Age： ______years Weight： ______Kg Height： ______cm
3. Nation： A. Han nationality B. Minority nationality
4. Educational level：

A. Secondary school and below B. Junior college

C. Undergraduate course D. Postgraduate or above

1. Marital status：

A. Unmarried B. Be married C. Divorced or widowed

1. Average monthly personal income (Unit: ¥)：

A. Less than 5000 B.5000-10000 C.10000-15000 D. More than 15,000

**Daily work**

1. Your primary place of work on a daily basis：

A. Outpatient service B. Emergency Department

C. Inpatient D. Laboratory

E. Radiology department F. Administrative office G. Others

1. Whether you work in a respiratory clinic or ward？

A. Yes B. No

1. Your main occupation type is：

A. Medical treatment B. Nursing work C. Medical skill D. Administration E. Work service F. Others：

1. The number of years you have been engaged in medical work：______ years,

Career years in current position：______ years.

1. Your average working hours per day：

A. Less than 8 hours B.8-12 hours C. More than 12 hours

1. Your average daily patient contact time：

A. Less than 4 hours B.4-8 hours C. More than 8 hours

1. Your current positional title level：

A. Senior Title B. Vice-senior Title

C. Middle title D. Primary title and below

**Personal protection practice**

1. What kind of protective equipment do you mainly use in your daily work？（Multiple choices）

A. Mask B. face mask C. Gloves D. Medical hat

E. Protective glasses F. Earplugs/earmuffs G. Protective suit

H. Shoe cover/protective shoe I. Others

1. Do you wear a mask in your daily work？（Select D to jump to 20）

A. Wear as required during all operations B. Sometimes forget to wear

C. Rarely wear D. Never wear

1. What type of mask do you wear in your daily work? （Select BCD to jump to 19）

A. N95 mask B. Surgical mask C. Disposable medical mask D. Others

1. If you wear an N95 mask, how do you wear it？

A. Wear continuously regardless of people/place B. For special people wear C. For special place wear

1. If you are wearing an N95 mask, have you done a compatibility test？

A. Yes B. No

1. How often do you wear and change your mask？

A. once a day B. A half-day C. Every 2-4 hours D. Less than 2 hours

1. What is your personal attitude towards wearing a mask：

A. Very necessary B. Passive acceptance C. Reject from the heart

1. The most commonly used air ventilation method in your work area：

A. Natural ventilation B. Mechanical ventilation (such as exhaust fan)

C. Central air conditioning ventilation D. Air purifier

1. The most common way to disinfect your work area：

A. Ultraviolet disinfection B. Circulating air UV air sterilizer

C. Electrostatic adsorption air sterilizer D. Chemical disinfection

F. Others________

1. Do you think it is necessary for the hospital to conduct regular training on occupational protection for all staff？

A. Yes B. No C. Not sure

1. How many times have you participated in training on the use of protective equipment or infection control in the past year?

A. Never B. Once C. Twice C. 3 times or more

1. Do you think there are occupational hazards in your daily work environment？

A. Yes B. No（jump to 26）

1. If yes, what do you think are the most serious occupational hazards：

A. Biological hazards B. Chemical hazards

C. Physical hazards D. Psychological hazards

1. Have you experienced occupational exposure at work？

A. Yes B. No

1. Are you familiar with the reporting process after occupational exposure？

A. Yes B. Not very familiar C. No

1. Have you ever suffered an injury at work？

A. Yes B. No（jump to 29）

1. The types of injuries or injuries that occur include（Multiple choices）：

A. Mechanical injury (acupuncture, sharp instrument injury, etc.)

B. Biological injury (skin infection, respiratory infection, blood infection, etc.)

C. Chemical injury (cytotoxic drugs, chemical clothing)

D. Physical injury (radiation injury, serious injury, etc.)

E. Psychological injury (occupational illness caused by stress overload, etc.)

F. Others

1. Whether to report to the department or unit sensitive control department after occupational exposure or injury？

A. Every time B. Report as appropriate C. Never

1. Do you think the unit attaches importance to the occupational exposure and occupational prevention and control of medical personnel？

A. Yes B. General attention

C. Pay little attention D. No

**Hand hygiene practice**

1. Do you know the World Health Organization hand hygiene "5" big opportunities?

A. No B. A little C. Yes

1. Do you think hand hygiene is important for nosocomial infection prevention and control?

A. Yes B. No

1. Do you think hand disinfection or wearing gloves can replace hand washing？ A. Yes B. No
2. Do you wash your hands before direct contact with patients？

A. Yes B. No

1. Do you wash your hands before cleaning and aseptic procedures？

A. Yes B. No

1. Do you wash your hands after direct contact with patients？

A. Yes B. No

1. Do you wash your hands after contact with the patient's body fluids and blood？ A. Yes B. No
2. Do you wash your hands after contact with the patient's surroundings？

A. Yes B. No

1. Do you wash your hands after removing gloves？

A. Yes B. No

1. How do you feel about hand hygiene in your daily work?

A. Full execution B. Most execution (around 80%)

C. Partial execution (around 50%) D. Little or no execution

1. If hand hygiene measures are not strictly implemented, the main reasons are:( Multiple choices)

A. Too busy with work B. Poor hand washing facilities

C. Wear gloves D. Hand sanitizer is not easily accessible

E. Not equipped with enough detergent near the sink

F. The effectiveness of hand washing or wiping is not effectively monitored

G. Not enough personal attention is paid to washing or wiping hands at work

H. Lack of hand washing or hand drying role models among colleagues

I. Cleansers and disinfectants irritate the skin and cause dryness

1. If you are the head of the department, do you think that the provision of rapid hand disinfectant will cost a lot of medical costs?

A. Yes B. No

1. Do you think that the incentive to wash hands will be promoted if there are incentives to wash hands or wipe hands?

A. Yes B. No

1. How about your participation in hand hygiene training in the past year?

A. Never B. Once

C. Twice D. 3 times or more

**Personal living habits**

1. Your annual influenza vaccination status:

A. Basic annual vaccination

B. Occasional vaccination (every two or three years)

C. Never or rarely vaccination

1. Have you been vaccinated against COVID-19？

A. No B. Incomplete vaccination

C. Complete vaccination D. Complete booster vaccination

1. Do you smoke？

A. Yes and still smoking B. Yes but quit C. No

1. If you have quit smoking, how long has it been since you last smoked (a cigarette, or even a puff)？

A. <1 mouth B. 1-6 mouths C. 6-12 mouths

D. 1-5years E. 5-10years F. ≥10 years

1. How much do you smoke per day when you smoke regularly？

A. Less than half a pack B. Half a pack

C. A pack D. Two packs E. More than two packs

1. How many smokers are there in your family______ people。
2. Do you drink alcohol？

A. No B. Occasional drink C. Regular drink D. Drink every day

1. How many liquor do you drink every week？

A. Less than two B.2-5 C.5-10 D. More than 10

1. Do you have any other medical conditions: (for each of the following: 1= yes, 2= no, 3= unknown)

A. Diabetes： ______________ B. Viral hepatitis： __________

C. COPD： _____________ D. Cancer： _______________

E. Cardiovascular and cerebrovascular diseases： _________ F. No above diseases

1. Did anyone in your household suffer from respiratory illness during the two weeks preceding the survey？

A. Yes，specific disease name ____________________ B. No

1. Have you had any symptoms of respiratory disease in the 1 month prior to this survey？

A. Yes B. No C. Nor sure

1. If you have symptoms related to respiratory diseases, what are the symptoms：

A. Cough B. Runny nose C. Sore throat

D. Myokymia E. Headache F. Fatigued G. Fever
